# Efficiency of copy number variation sequencing combined with karyotyping in fetuses with congenital heart disease and the following outcomes

**DOI:** 10.1186/s13039-024-00681-5

**Published:** 2024-05-13

**Authors:** Xuezhen Wang, Jing Sha, Yu Han, Min Pang, Min Liu, Mengna Liu, Bei Zhang, Jingfang Zhai

**Affiliations:** 1 Department of Prenatal Diagnosis Medical Center, Xuzhou Central Hospital, Xuzhou Clinical School of Xuzhou Medical University, Jiefang South Road No.199, Xuzhou, 221009 Jiangsu China; 2 Graduate School of Bengbu Medical University, Donghai Avenue No.2600, Bengbu, 233000 Anhui China; 3 Key Laboratory of Brain Diseases Bioinformation of Xuzhou Medical University, Tongshan Road No.209, Xuzhou, 221004 Jiangsu China

**Keywords:** Congenital heart diseases, Genetic etiology, Karyotype analysis, Copy number variation sequencing

## Abstract

**Background:**

Both copy number variant-sequencing (CNV-seq) and karyotype analysis have been used as powerful tools in the genetic aetiology of fetuses with congenital heart diseases (CHD). However, CNV-seq brings clinicians more confusions to interpret the detection results related to CHD with or without extracardiac abnormalities. Hence, we conducted this study to investigate the clinical value of CNV-seq in fetuses with CHD.

**Results:**

A total of 167 patients with fetal CHD including 36 single CHD (sCHD), 41 compound CHD (cCHD) and 90 non-isolated CHD (niCHD) were recruited into the study. 28 cases **(**16.77%, 28/167) were revealed with chromosomal abnormalities at the level of karyotype. The pathogenic detection rate (DR) of CNV-seq (23.17%, 19/82) was higher than that of karyotyping (15.85%, 13/82) in 82 cases by CNV-seq and karyotyping simultaneously. The DR of pathogenic copy number variations (PCNVs) (31.43%) was higher in niCHD subgroup than that in sCHD and cCHD (9.52% and 23.08%). Conotruncal defect (CTD) was one of the most common heart malformations with the highest DR of PCNVs (50%) in 7 categories of CHD. In terms of all the pregnancy outcomes, 67 (40.12%) cases were terminated and 100 (59.88%) cases were live neonates. Only two among 34 cases with a pathogenic genetic result chose to continue the pregnancy.

**Conclusions:**

CNV-seq combined with karyotyping is a reliable and accurate prenatal technique for identifying pathogenic chromosomal abnormalities associated with fetal CHD with or without extracardiac abnormalities, which can assist clinicians to perform detailed genetic counselling with regard to the etiology and related outcomes of CHD.

**Supplementary Information:**

The online version contains supplementary material available at 10.1186/s13039-024-00681-5.

## Background

Congenital heart disease (CHD) is the most common birth defect, occurring in approximately 0.4–5% of live births [1, 2], and up to 10% of stillbirths [3]. Genetic abnormalities, including aneuploidies (AUP), copy number variants (CNVs) and single nucleotide variants, play a significant role in determining the clinical outcome of CHD [4, 5]. With surgical development, most types of CHD return to normal or near normal after cardiovascular surgery. Once combined with chromosomal abnormalities, the prognosis of fetuses with CHD is poor due to common complications such as severe extracardiac structural abnormalities, mental retardation and developmental delay, etc. Therefore, prenatal genetic diagnosis is strongly recommended for fetuses with CHD. Compared to traditional G-banding karyotyping, CNV-seq can detect additional CNVs of more than 100 kb [6, 7]. Both of the complementary techniques may improve the detection rate (DR) of chromosomal abnormalities in CHD fetuses. However, the drawback of present condition is that CNV-seq detection brings more results for clinicians to interpret the detection results related with the fetal manifestations. Hence, it would of difficulty to get a deeper insight to understand the genetic abnormalities associated with fetuses with CHD through CNV-seq combined with karyotyping. Herein, our group conducted a retrospective study to evaluate the clinical value in terms of the genetic etiology by CNV-seq combined with karyotyping and ultimate pregnancy outcomes of fetuses with CHD. In addition, we also stratified 82 cases by simultaneous CNV-seq and karyotype detection to better understand the DR of chromosomal abnormalities in different types of CHD, and compared the frequency of chromosomal abnormalities in fetuses with single CHD (sCHD), compound CHD (cCHD) and non-isolated CHD (niCHD).

## Results

### Basic characteristics of study subjects

From January 2016 to October 2022, a total of 167 pregnant women with fetal CHD including 36 sCHD, 41 cCHD and 90 niCHD were recruited into the study. Although there were no significant differences in maternal age (MA), gestational age (GA) and parity history (*P* > 0.05) among sCHD, cCHD, and niCHD groups, a significant difference of pregnancy outcome (*P* < 0.01) suggested that the fetuses with niCHD were more likely to be terminated. In the end, 100 cases (59.88%) chose to continue their pregnancy and 67 cases (40.12%) chose to terminate their pregnancy (Table 1).

**Table 1:** Basic characteristics of study subjects

Characteristics	Isolated CHD		Non-isolated CHD	χ^2^	*P*
	Single CHD	Compound CHD			
Mean MA	28.57 ± 3.80	29.93 ± 4.30	28.28 ± 4.34		
< 35	34	33	82	4.267	0.118
≥ 35	2	8	8		
Mean GA(W)	24.83 ± 3.03	24.64 ± 2.86	23.45 ± 2.78		
Parity history					
Nulliparous	13	16	46	3.099	0.212
Parous	23	25	44		
Pregnancy outcomes					
Delivery	34	17	49	29.807	0.000
TOP*	2	24	41		
Total	36	41	90		

*TOP, termination of pregnancy; GA, gestational age; MA, maternal age

### Karyotype and CNV-seq results in CHD cases

21 AUP and 7 unbalanced chromosomal structural abnormalities were found (Table 2) in 167 fetuses. 21 AUP were consisted of 9 trisomy 21, 7 trisomy 18, 1 trisomy 13, 1 partial trisomy 14 and 9 (47,XY, + 14,der(14) (9;14)(9p23;q22)mat), 2 monosomy X (containing 1 mosaic monosomy X) and 1 47, XXY, demonstrating that trisomy 21 and 18 accounted for the major proportion. The DR of chromosomal abnormalities in the niCHD subgroup (23.33%, 21/90) were obviously higher than that in sCHD group (2.78%, 1/36) (*P* = 0.013).

**Table 2:** Chromosomal aberration in 167 fetal CHD detected by karyotyping

Chromosomal aberration		Single CHD	Compound CHD	Non-isolated CHD	*P*
n		36	41	90	
Numerical abnormalities	T21	1	3	5	0.652
	T18	0	1	6	0.266
	T13	0	0	1	1.000
	Other autosomes	0	0	1	1.000
	sex chromosome	0	0	3	0.421
Structural abnormalities		0	2	5	0.507
Total		1	6	21	0.005

In addition, in 82 cases of copy number variation detection combined with karyotype analysis, the pathogenic DR of CNV-seq (23.17%, 19/82) was higher than that of karyotype analysis (15.85%, 13/82) (*P* = 0.237) (Table 3). The 31.43% (11/35) DR of pathogenic copy number variations (PCNVs) in the niCHD group was higher than 9.52% (2/21) in the sCHD and 23.08% (6/26) in the cCHD, with no significant difference in the DR of chromosomal aberrations (*P* = 0.156) or in the PCNVs among the three groups (*P* = 0.142) (Table 3). Moreover, CHD cases were classified to 7 categories according to international statistical classification of diseases and related health problems 10th revision (ICD-10), and the results showed that the DR of PCNVs (50%, 7/14) in conotruncal defect (CTD) is highest, followed by atrioventricular septal defect (AVSD) (Additional file 1: Table S1). Thirty CNVs of 82 cases [19 PCNVs, 6 benign (B) CNVs, 2 like benign (LB) CNVs, and 3 variants of uncertain significance (VOUS)] were revealed by CNV-seq, including several syndromes such as Down's syndrome (n = 4), Edward's syndrome (n = 2), 22q11.2 microdeletion syndrome (n = 1), 1p36 microdeletion syndrome (n = 1), Wolf-Hirschhorn syndrome (n = 1), and recurrent Simpson-Golabi-Behmel syndrome type 1 (SGBS1) (n = 2) (Table 4), plus extra CHD anomalies indicating related syndromes should be considered by clinicians once facing multiple fetal structural abnormalities.

**Table 3:** Abnormal results in 82 CHD detected by karyotyping and CNV-seq

Category	total	chromosomal aberration (%)	PCNVs(%)
Single CHD	21	1(4.76%)	2(9.52%)
Compound CHD	26	4(15.38%)	6(23.08%)
Non-isolated CHD	35	8(22.86%)	11(31.43%)
total	82	13(15.85%)	19(23.17%)
χ^2^		3.713	3.905
*P*		0.156	0.142

PCNVs, pathogenic copy number variations

**Table 4:** Phenotype and genotype list of CNVs

Cases	MA	GA	Cardiac defects	Extra cardiac defects	karyotype	CNVs	Size	Categories	Related syndromes	Outcomes	gravidity	Parity
1	24	24 + 3	PLSVC,DCS	+	46,XY	dup(11)(p11.12)	0.8 Mb	B	/	NL	3	0
2	35	27 + 6	PS, Mild TR, Tortuous DA	–	46,XY	del(14)(q11.2)	0.64 Mb	B	/	NL	3	1
3	29	27 + 1	VSD	–	46,XY	del(14)(q11.2)	0.46 Mb	B	/	NL	4	1
4	26	26 + 2	R-AA, R-DA	–	46,XX	del(14)(q11.2q11.2)	0.60 Mb	B	/	NL	3	0
5	29	24	single atrium, mild MR, PLSVC	–	46,XY	del(14)(q11.2)	0.44 Mb	B	/	TOP	4	2
6	26	22 + 3	TOF, R-AA; L-ASA	–	46,XY,15pstk +	del(11)(p14.3p14.3)	0.36 Mb	B	/	TOP	1	0
7	27	28 + 4	C-shaped vascular ring, L-AA, L-DA, R-ASA, L-EIF	–	46,XX	del(9)(p23)	0.22 Mb	LB	/	NL	3	1
8	30	28	VSD(0.6 mm)	–	46,XX	dup(8)(p23.1)	0.3 Mb	LB	/	NL	3	1
9	34	24 + 1	AVSD	–	46,XX	dup(8)(p21.3p21.2)	1.58 Mb	VOUS	/	TOP	4	1
10	29	25 + 6	R-AA, Mirror branch of BA	+	46,XY,1qh +	dup(X)(p21.22p21.1)	0.24 Mb	VOUS	/	TOP	4	1
11	29	23 + 4	SV,PTA	+	47,XX, + mar	dup(4)(q11q13.1)	10.64 Mb	VOUS	/	TOP	5	1
12	28	24 + 3	VSD(2.9 mm),Increased ratio of lung to main body,Aortic stenosis and disconnection,DIVC	–	46,XX,der(11)t(7;11)(q22;q25)pat	dup(7)(q21.3q36.3); del(11)(q24.3q25)	61.90 Mb; 4.68 Mb	P; LP	Polydactyly, Preaxial II	TOP	2	0
13	30	27 + 3	PLSVC	+	46,XX,der(6) t(6;10)(p25;p13)	del(6)(p25.3p25.1); dup(10)(p15.3p13)	6.32 Mb; 13.94 Mb	P; VOUS	Axenfeld-Rieger syndrome type 3	TOP	1	0
14	30	24 + 6	Aortic stenosis and disconnection	+	46,XX,del(1)(p36)	del(1)(p36.33p36.22)	8.7 Mb	p	1p36 microdeletion syndrome	TOP	3	1
15	33	28 + 5	VSD(1.2 mm),R-AA,L-ASA	–	46,XX	del(3)(q26.33q27.3)	5.08 Mb	P	/	TOP	3	1
16	26	21 + 6	PLSVC	+	46,XX,del(4)(p15.3)	del(4)(p16.3p15.31)	21 Mb	P	Wolf-Hirschhorn syndrome	TOP	1	0
17	24	26 + 3	VSD(3 mm),Partial TGA, DOTR	+	46,XY	del(X)(q26.2)	0.26 Mb	P	Simpson-Golabi-Behmel syndrome	TOP	1	0
18	33	23	TOF	+	46,XY,der(16)t(7:16)(p14.1;p13.3)pat	del(16)(p13.3p13.3); dup(7)(p22.3p14.1)	1.46 Mb; 37.5 Mb	P; P	Alpha thalassaemia mental retardation—16	TOP	1	0
19	27	23 + 2	AVSD,CAV,PCFO, ,Enlarged right heart	+	46,XX	del(16)(q24.1q24.2)	2.12 Mb	P	Alveolar capillary dysplasia with misalignment of pulmonary veins, Lymphedema–distichiasis syndrome	TOP	2	1
20	25	17 + 1	VSD(1.1 mm)	+	46,XY	del(X)(q26.2)	0.26 Mb	P	Simpson-Golabi-Behmel syndrome	TOP	2	0
21	39	24 + 3	VSD(1.2 mm),mild TR	+	47,XX, + 21	47,XX, + 21	33.83 Mb	P	Down’s syndrome	TOP	2	1
22	24	25 + 3	VSD(1.2 mm),mild TR	–	47,XY, + 21	47,XX, + 21	33.83 Mb	P	Down’s syndrome	TOP	2	1
23	29	27	VSD(4.2 mm)	+	47,XX, + 18	47,XX, + 18	78.08 Mb	P	Edward’s syndrome	TOP	2	1
24	30	19 + 2	VSD,thinner aorta	–	46,XX,add(7)(?::p22 → qter)	del(7)(p22.3p22.2); dup(16)(q22.1q24.3)	3.86 Mb; 20.96 Mb	P; P	/	TOP	4	1
25	35	17 + 2	VSD,TOF,R-AA	–	46,XY	del(22)(q11.21q11.2)	2.60 Mb	P	22q11.2 deletion syndrome	TOP	8	2
26	36	18 + 6	VSD, overriding aorta	–	47,XX, + 21	47,XX, + 21	33.83 Mb	P	Down’s syndrome	TOP		
27	31	19 + 2	VSD	–	46,XY,rob(14;21), + 21	dup(21)(q11.12q22.3)	33.8 Mb	P	Down’s syndrome	TOP	1	0
28	30	19 + 1	TOF	+	46,XY,add(13)(q31)	del(13)q(33.2q34); dup(8)(q22.2q24.3); dup(6)(q26)	9.54 Mb; 45.26 Mb; 0.38 Mb	P; P; VOUS	/	TOP	1	0
29	27	18 + 5	VSD	–	46,XY,16qh +	dup(17)(p12); dup(15)(q13.3)(maternal)	1.34 Mb; 0.42 Mb	P; B	Hereditary neuropathy with liability to pressure palsies; Charcot-Marie-Tooth disease-IA	NL	2	1
30	38	20 + 5	VSD	+	47,XY, + 18	trisomy 18		P	Edward’s syndrome	TOP	4	2

AA, aortic arch; ASA, aberrant subclavian artery; AVSD, atrioventricular septal defect; B, benign; BA, brachiocephalic artery; CAV, common atrioventricular valve; DA, ductus arteriosus; DCS, dilated coronary sinus; DIVC, double inferior vena cava; DORT, double-outlet right ventricle; DPA, dilated pulmonary artery; EIF, echogenic intracardiac focus; GA, gestational week; L, left; LB, likely benign; LP, likely pathogenic; MA, maternal age; MR, mitral regurgitation; NL,normal labor; P, pathogenic; PCFO, premature closure of foramen ovale; PLSVC, persistent left superior vena Cava; PS, pulmonary stenosis; PTA, persistent truncus arteriosus; R, right; SV, single ventricle; TGA, transposition of great artery; TOF, tetralogy of fallot; TOP, termination of pregnancy; TR, tricuspid regurgitation; VSD, ventricular septal defect; VOUS, variants of uncertain significance

Among 35 niCHD cases, CHD with cardiovascular structural anomalies and cardiovascular soft index anomalies accounted for 60% (21/35) and 40% (14/35), respectively. Although the DR of PCNVs in the former group (38.1%, 8/21) was higher than that in the latter group (21.43%, 3/14), there were no statistical significance between two groups (*P* = 0.623) (Additional file 2: Table S2).

### Pregnancy outcomes and prognosis classification of all cases

Compared to fetuses without CNVs or with B/LB/VOUS CNVs, more couples chose to induce labor regarding to their fetuses with AUP or P/LP CNVs (94.12% (32/34) vs. 26.32% (35/133), *P* < 0.001) (Additional file 3: Table S3). 133 fetuses without pathological chromosomal alterations were classified into four grades according to the prognostic grading of fetal CHD (Additional file 4: Table S4) [8], and we found fetuses with CHD of grade III and IV were all terminated. Overall, thirty-five (26.32%, 35/133) were terminated and ninty-eight (73.68%, 98/133) were live neonates in the following day. The fetuses with chromosomal anomalies who were terminated included VOUS [n = 3, one case with a 1.58-Mb 8p21.3p21.2 duplication presented with AVSD, one case with a 0.24-Mb X p21.22 duplication manifested right aortic arch (R-AA), mirror branch of brachiocephalic artery (BA) and hydrocephalus, and one case with a 10.64-Mb 4q13.1 duplication showed single ventricle (SV), persistent truncus arteriosus (PTA) and single umbilical artery], BCNVs [n = 2, one case with a 0.44-Mb 14q11.2 deletion presented with single atrium, mild mitral regurgitation (MR), persistent left superior vena cava (PLSVC), and one case with a 0.36-Mb 11p14.3 deletion showed tetralogy of fallot (TOF), R-AA, left aberrant subclavian artery (L-ASA)]. Other 30 cases chose to terminate the pregnancy due to poor prognosis of severe cardiac defects (with grade 3 and 4) or severe extracardiac defects (with grade 1 and 2).

In the six-month follow-up studies of 100 newborns, including 7 cases with prenatal ventricular/atrial septal defect (VSD/ASD) showed normal cardiovascular structure on ultrasound, 5 cases showed new ASD or more severe VSD than before, 77 newborns were consistent with prenatal cardiovascular structure on ultrasound, 11 newborns without further postpartum cardiac ultrasound examination (two fetuses with pathogenic results were included, and presented normal). Hence, dynamic ultrasound evaluation and follow-up after birth could provide practical guidance to clinicians.

## Discussion

Previous study has demonstrated that the majority of CHD and other extracardiac defects may result from genetic factors, among which AUP accounts for 9–18% of CHD [4, 9, 10]. In our present paper, 28 cases (16.77%) of chromosomal aberrations were detected by karyotyping in 167 fetuses with CHD. The DR of aneuploidies accounted for 12.57% (21/167) in our study, among which trisomy 21 and 18 presented with the largest proportion (76.19%, 16/21), similar results to those in the literature [11–13]. The above information indicates that the dose alterations of genetic materials might increases the risk of malformations of fetuses [12]. Hence, in clinical practice, dynamic ultrasound should be supervised to evaluate systematic and comprehensive structural investigation for the early revealed imbalanced chromosome number or structural alterations. Moreover, 3% to 25% of fetuses with CHD have been reported to be associated with PCNVs [9, 14, 15]. As shown in Table 3, the pathogenic DR of CNV-seq was 23.17% (19/82) similar to that observed in the previous study [13], containing severe syndromes, such as SGBS1, 22q11.2 microdeletion. Relative to traditional karyotyping, an additional 7.32% PCNVs (6/82) by CNV-seq in our study was similar to previous research 7.70 ~ 7.95% by chromosome microarray analysis (CMA) [12, 16]. Hence, CNV-seq could provide an efficient and equivalent to CMA and recognize the microdeletion or microduplication of chromosomes (MMS). Besides above advantages, CNV-seq based on next-generation sequencing is emerging as an alternative methodology due to needed smaller sample size of test, faster experimental cycle and lower cost [11, 17, 18].

It is worth noting that the majority of the CHD fetuses with the MMS were characterized by multiple-system structural anomalies (Table 4) [19]. As can been seen from the basic characteristics of our subjects, fetuses with CHD had no association with MA, GA and parity history (Table 1). In line with the previous report, MMS has been confirmed to be unrelated to the age of the pregnant women [20] Therefore, further comprehensive genetic assessment is required for structural abnormalities of fetuses [21, 22]. Therefore, prenatal diagnosis is strongly recommended for fetuses with CHD whether accompanied with other systematic structure malformations. It is vital for clinicians to comprehensively manage fetuses with CHD based on chromosomal abnormalities, phenotypes in ultrasonic and clinicians’ recommendation.

In 7 subgroups of fetuses with CHD, the statistical analysis demonstrated the major frequencies of chromosomal abnormalities occurred in fetuses with CTD (7/14, 50%), followed by AVSD (1/3, 33.33%), septal defects (6/23, 26.09%) (Additional file 1: Table S1). These DR of chromosomal abnormalities in 7 subgroups were not consistent with the previous reported (73.7% in AVSD, 25.7% in CTD, 17.5% in septal defects) [12], as might be related to the sample sizes and proportions of different types of CHD. The different degrees of DR of chromosomal abnormalities were found in all 7 group, suggesting that fetuses with CHD during pregnancy require chromosome diagnosis. Moreover, 26 fetuses with cCHD presented with 23.08% (6/26) PCNVs including related to 3 kinds of syndromes, significantly higher than those with sCHD 9.52% (2/21) (Table 3), indicating pathogenic CNVs associated syndromes should be considered for compound CHD. In addition, the DR of PCNVs was significantly higher in niCHD with structural anomalies (38.1%, 8/21) than that in niCHD with soft index anomalies (21.43%, 3/14) (Additional file 2: Table S2), consistent with previous studies indicating that CHD fetuses with structural abnormalities are more likely to be related to genetic disorders [23, 24]. Hence, more attentions should be paid to fetuses with multiple structural abnormalities besides CHD.

Once a fetus is diagnosed with CHD, dynamic comprehensive ultrasound, echocardiograms and genetic assessments should be performed properly. Only in this way, could the clinician offer more useful counseling and help for the cases. In our study, there were only 2 normal deliveries in 34 cases with pathogenic genetic results, one fetus with 47, XXY, the other with a 1.34-Mb duplication on chromosome 17p12 inherited from his mother, showing that genetic factors have a significant impact on outcomes of fetuses with CHD. CHD fetuses with chromosomal abnormalities may have a significantly increasing risk of mortality, and require more medical care and medication. Of the remaining 133 cases without pathogenetic chromosomes, 35 cases underwent labor inductions, including 2 BCNVs (1 case with single atrium, mild MR, PLSVC; 1 case with TOF, R-AA; L-ASA), 3 VOUS (1 case with AVSD, 1 case with R-AA, Mirror branch of BA and hydrocephalus, 1 case with SV, PTA and single umbilical artery), suggesting that genetic factors as one aspect to merely be considered and CHD need to be comprehensively evaluated in conjunction with clinical phenotypes (Additional file 4: Table S4). After searching relevant literatures, we have not found the same mutation as VUS fetuses in our report. More data need to be confirmed the relationship between phenotype and VOUS CNVs. For pregnant women who continued their pregnancy, the usual obstetric follow-up of maternal health and the specialized follow-up of fetal health (regular morphological ultrasound) should be ensured. Hence, the destiny of fetuses with CHD often depends on the chromosomal aberrations, the severity of cardiac/extracardiac defects and cognitive level of the parents. It is worth noting that two similar boy fetuses with CHD diagnosed as SGBS1 recurred in a family, and CNV-seq verified it from the mother. Based on SGBS1 as an X-linked recessive disorder, preimplantation genetic diagnosis (PGD) was recommended for the next pregnancy [25, 26]. Therefore, it is helpful to clear genetic history and contribute to the reasonable guidance of second pregnancy.

Although CNV-seq is a very valuable detection tool for prenatal diagnosis, it also has limitations. It cannot effectively measure chromosomal inversion, balanced translocation or some special triploids etc., however, karyotype analysis can efficiently complement the above results. Therefore, the combined application of the two technologies can effectively increase the DR of chromosome abnormalities. VOUS is an additional finding of CNV-Seq to detect fetal CHD, and the application of CNV-Seq technology in prenatal diagnosis of fetal CHD brings the biggest clinical difficulty, which is the interpretation of VOUS [27].

## Conclusion

The occurrence of CHD is related to chromosomal AUP and CNVs. CNV-seq is an effective adjunct to traditional chromosomal karyotyping. Genetic analysis combined with dynamic ultrasound screening and multidisciplinary counselling can effectively provide valuable information to the clinicians and patients.

## Methods

### Study subjects

This retrospective study was performed in the prenatal diagnosis center of Xuzhou Central Hospital of Jiangsu Province from January 2016 to October 2022. 167 fetuses with CHD by echocardiogram were enrolled in our study. Written informed consents for data collection and manuscript publication were provided by all the couples. The demographic characteristics were recorded through a comprehensive questionnaire including maternal age (MA), gestational week (GA), pregnancy outcomes, gravidity, parity, histories of abnormal pregnancy, as well as hereditary diseases in Table 1. The average MA was 28.75 ± 4.27 years, the mean GA was 24.05 ± 2.93 weeks; nulliparas accounted for 45.51% and multiparas accounted for 54.49%. Of 167 fetuses with CHD, isolated CHD (n = 77) included sCHD (n = 36) and cCHD (n = 41), and niCHD (n = 90) had additional extracardiac defects according to the classification of fetal CHD. All pregnant women accepted invasive prenatal diagnosis (127 amniocenteses and 40 cordocentesis) for karyotyping, among whom 82 underwent CNV-seq simultaneously. And all subjects denied hereditary diseases. The proposal for our study has been approved by the ethics committee of Xuzhou Central Hospital (XZXY-LK-20230314-034).

### Karyotype analysis

The obtained amniotic fluid and umbilical cord blood were cultured and stained with G-banding according to the general operating procedures. 20 well-dispersed, medium-length metaphase phases from each specimen were analyzed according to ISCN (2016,2020) criteria [28, 29], when mosaicism or abnormal karyotypes were found, this was increased to 100 metaphase phases.

### CNV-seq

Genomic DNA (gDNA) was extracted from amniotic fluid or fetal cord blood. Then 50 ng of DNA was fragmented and DNA libraries were constructed by end repair, ligated with sequencing adaptors, polymerase chain reaction (PCR) amplifification, and DNA libraries were subjected to massively parallel sequencing using NextSeq 500 platform (Illumina, San Diego, USA), to generate approximately 5 million raw sequencing reads with genomic DNA sequences of 36 base pair in length. Using the hg19 genomic sequence as reference, a total of 2.8–3.2 million reads were uniquely and precisely mapped using the BurrowseWheeler algorithm [30]. Mapped reads were allocated progressively to 20- kilobase (kb) bin sizes from the p to q arms of the 24 chromosomes. And the criteria of sequencing copy number (SCN) results refered to the attached Additional file 5: Table S5. Several public databases such as Database of Genomic Variants (DGV), Online Mendelian Inheritance in Man (OMIM), DECIPHER, University of California, etc., were utilized to interpret the results as gains and losses of copy number. CNVs were interpreted and divided into five categories: pathogenic(P), likely pathogenic(LP), VOUS, likely benign(LB), and benign(B), according to the guidelines outlined by the American College of Medical Genetics (ACMG) [7].

### Statistical analysis

The data were analyzed by SPSS software (version 26.0, IBM, Armonk, NY, USA). Continuous variables were expressed as mean ± standard deviation (SD). Enumeration data were expressed as frequency, and chi-square test or Fisher's exact test was used to compare the rates between groups (*P* < 0.05) was regarded as statistically significant).

### Supplementary Information

**Additional file 1**.

**Additional file 2**.

**Additional file 3**.

**Additional file 4**.

**Additional file 5**.

## Data Availability

Not applicable.

## Abbreviations

ASD: Atrial septal defect
AUP: Aneuploids
AVSD: Atrioventricular septal defect
B: Benign
BA: Brachiocephalic artery
CNV-Seq: Copy number variant sequencing
CHD: Congenital heart disease
CMA: Chromosomal microarray analysis
CNVs: Copy number variants
CTD: Conotruncal defects
DR: Detection rate
GA: Gestational week
ICD-10: International statistical classification of diseases and related health problems 10th revision
L-ASA: Left aberrant subclavian artery
LB: Likely benign
LP: Likely pathogenic
LVOTD: Left ventricular outflow tract defects
MA: Maternal age
MMS: Microduplication of chromosomes
MR: Mitral regurgitation
P: Pathogenic
PLSVC: Persistent left superior vena cava
PTA: Persistent truncus arteriosus
R-AA: Right aortic arch
RVOTD: Right ventricular outflow tract defects
SGBS1: Simpson-golabi-behmel syndrome type 1
TOF: Tetralogy of fallot
TOP: Terminate the pregnancy
VOUS: Variation of uncertain significance
VSD: Ventricular septal defect

## References

[1] van der Linde D, Konings EE, Slager MA (2011). Birth prevalence of congenital heart disease worldwide: a systematic review and meta analysis. J Am Coll Cardiol.

[2] Qu Y, Liu X, Zhuang J (2016). Incidence of congenital heart disease: the 9-year experience of the Guangdong registry of congenital heart disease, China. PLoS ONE.

[3] Yan Y, Wu Q, Zhang L (2014). Detection of submicroscopic chromosomal aberrations by array based comparative genomic hybridization in fetuses with congenital heart disease. Ultrasound Obstetr Gynecol.

[4] Zaidi S, Brueckner M (2017). Genetics and genomics of congenital heart disease. Circ Res.

[5] Mone F, Eberhardt RY, Morris RK (2021). Congenital heart disease and the diagnostic yield with exome sequencing (CODE) study: prospective cohort study and systematic review. Ultrasound Obstet Gynecol.

[6] Mustafa HJ, Jacobs KM, Tessier KM (2020). Chromosomal microarray analysis in the investigation of prenatally diagnosed congenital heart disease. Am J Obstet Gynecol MFM.

[7] Riggs ER, Andersen EF, Cherry AM (2020). Technical standards for the interpretation and reporting of constitutional copy-number variants: a joint consensus recommendation of the American college of medical genetics and genomics (ACMG) and the clinical genome resource (ClinGen). Genet Med.

[8] Prognosis Grading and Perinatal Risk Assessment of Fetal Heart Disease. Expert Panel. Consensus on the medical model and technical process of multidisciplinary diagnosis and treatment and precision integrated prevention and management of fetal heart disease in maternal-fetal medicine (Part I): Expert consensus on prognosis grading and perinatal risk assessment of fetal heart disease. Chin J Perinat Med. 2022;25(5):321–325.

[9] Cowan JR, Ware SM (2015). Genetics and genetic testing in congenital heart disease. Clin Perinatol.

[10] Song MS, Hu A, Dyamenahalli U (2009). Extracardiac lesions and chromosomal abnormalities associated with major fetal heart defects: comparison of intrauterine, postnatal and postmortem diagnoses. Ultrasound Obstet Gynecol.

[11] Zhu X, Li J, Ru T (2016). Identification of copy number variations associated with congenital heart disease by chromosomal microarray analysis and next-generation sequencing. Prenat Diagn.

[12] Wang Y, Cao L, Liang D (2018). Prenatal chromosomal microarray analysis in fetuses with congenital heart disease: a prospective cohort study. Am J Obstetr Gynecol.

[13] Yi T, Hao X, Sun H (2023). Genetic aetiology distribution of 398 foetuses with congenital heart disease in the prenatal setting. ESC Heart Fail.

[14] van Nisselrooij AEL, Lugthart MA, Clur SA (2020). The prevalence of genetic diagnoses in fetuses with severe congenital heart defects. Genet Med.

[15] Ehrlich L, Prakash SK (2022). Copy-number variation in congenital heart disease. Curr Opin Genet Dev.

[16] Hureaux M, Guterman S, Hervé B (2019). Chromosomal microarray analysis in fetuses with an isolated congenital heart defect: A retrospective, nationwide, multicenter study in France. Prenat Diagn.

[17] Fu F, Li R, Yu QX, Dang X, Yan SJ, Zhou H, Cheng K, Huang RB, Wang Y, Zhang YL, Jing XY, Zhang LN, Li DZ, Liao C (2022). The value of a comprehensive genomic evaluation in prenatal diagnosis of genetic diseases: a retrospective study. Genes.

[18] Rodriguez-Revenga L, Madrigal I, Borrell A, Martinez JM, Sabria J, Martin L, Jimenez W, Mira A, Badenas C, Milà M (2020). Chromosome microarray analysis should be offered to all invasive prenatal diagnostic testing following a normal rapid aneuploidy test result. Clin Genet.

[19] Mohammadzadeh A, Akbaroghli S, Aghaei-Moghadam E (2019). Investigation of chromosomal abnormalities and microdeletion/microduplication(s) in fifty Iranian patients with multiple congenital anomalies. Cell J.

[20] Wapner RJ, Babiarz JE, Levy B (2015). Expanding the scope of noninvasive prenatal testing: detection of fetal microdeletion syndromes. Am J Obstet Gynecol.

[21] Luo SY, Meng DH, Li QF (2018). Genetic testing and pregnancy outcome analysis of 362 fetuses with congenital heart disease identified by prenatal ultrasound. Arq Bras Cardiol.

[22] Jansen FA, Blumenfeld YJ, Fisher A (2015). Array comparative genomic hybridization and fetal congenital heart defects: a systematic review and meta-analysis. Ultrasound Obstet Gynecol.

[23] Wang M, Xiao JP, Zhao L (2022). Clinical value of chromosome microarray analysis in the etiological diagnosis of fetuses with congenital heart disease. Chinese J Pract Gynecol Obstetr.

[24] Donnelly JC, Platt LD, Rebarber A (2014). Association of copy number variants with specific ultrasonographically detected fetal anomalies. Obstet Gynecol.

[25] Cirillo A, Lioncino M, Maratea A, et al. Clinical manifestations of 22q11.2 deletion syndrome. Heart Fail Clin. 2022;18(1):155–164. 10.1016/j.hfc.2021.07.009 34776076

[26] Sha J, Tan FF, Zhai JF, et al. A prenatal case of Simpson-Golabi-Behmel syndrome type 1 with a 0.26-Mb deletion fragment at Xq26.2 inherited from mother: case report. Medicine (Baltimore). 2022;101(16):e29222. 10.1097/MD.0000000000029222 PMC9276221 35482990

[27] Clinical genetics group of medical genetics branch Chinese medical association, professional committee for prenatal diagnosis of genetic diseases medical genetics branch of Chinese medical association, group of genetic disease prevention and control birth defect prevention and control committee of Chinese society of preventive medicine. Zhonghua Yi Xue Yi Chuan Xue Za Zhi. 2019;36(4):293-296. 10.3760/cma.j.issn.1003-9406.2019.04.001 30950010

[28] ISCN 2016: An International System for Human Cytogenomic Nomenclature (2016). McGowan-Jordan J, Simons A, Schmid M, eds. Basel: Karger, 2016; also in Cytogenet Genome Res, 2016;149:1–140.

[29] ISCN 2020: An international system for human cytogenomic nomenclature (2020). McGowan-Jordan J, Ros JH, Sarah M, eds. Basel: Karger, 2020; also in Cytogenet Genome Res, 2020;160:341–503.

[30] Qiao J, Yuan J, Hu W (2022). Combined diagnosis of QF-PCR and CNV-Seq in fetal chromosomal abnormalities: a new perspective on prenatal diagnosis. J Clin Lab Anal.

