# Supplementary material for: Efficiency of copy number variation sequencing combined with karyotyping in fetuses with congenital heart disease and the following outcomes
Source: Mol Cytogenet. 2024 May 13;17:12. doi: 10.1186/s13039-024-00681-5 (PMC11089693; doi:10.1186/s13039-024-00681-5)
Supplement: Supplementary file 1 — Additional file 1. [file 13039_2024_681_MOESM1_ESM.docx]

| Table S1 CNVs in 82 fetal CHD detected by CNV-seq | | | |
| --- | --- | --- | --- |
| Category |  | CNVs | |
|  | Total | P (%) | VOUS (%) |
| CTD | 14 | 7 (50.00%) | 0 (0.00%) |
| AVSD | 3 | 1 (33.33%) | 1 (33.33%) |
| LVOTD | 6 | 1 (16.67%) | 0 (0.00%) |
| RVOTD | 9 | 2 (22.22%) | 0 (0.00%) |
| Septal defect | 23 | 6 (26.09%) | 0 (0.00%) |
| Others | 27 | 2 (7.41%) | 2 (7.41%) |
| Total | 82 | 19 (23.17%) | 3 (3.66%) |
| CTD, conotruncal defects; AVSD, atrioventricular septal defect; LVOTD, left ventricular outflow tract defects; RVOTD, right ventricular outflow tractdefects; Septal defect, atrial /ventricular septal defect | | | |
