# Supplementary material for: Efficiency of copy number variation sequencing combined with karyotyping in fetuses with congenital heart disease and the following outcomes
Source: Mol Cytogenet. 2024 May 13;17:12. doi: 10.1186/s13039-024-00681-5 (PMC11089693; doi:10.1186/s13039-024-00681-5)
Supplement: Supplementary file 2 — Additional file 2. [file 13039_2024_681_MOESM2_ESM.docx]

| Table S2 Abnormal karyotype and CNV-seq results in 35 non-isolated CHD | | | | | |
| --- | --- | --- | --- | --- | --- |
| Extracardiac abnormality |  | Karyotype results | | CNV-seq results | |
|  | Total | Chromosomal aberration | DR(%) | PCNVs | DR(%) |
| Structural | 21 | 6 | 28.57 | 8 | 38.10 |
| Soft index | 14 | 3 | 21.43 | 3 | 21.43 |
| χ2 |  | 0.006 | | 0.241 | |
| *P* |  | 0.937 | | 0.623 | |
