# Supplementary material for: Efficiency of copy number variation sequencing combined with karyotyping in fetuses with congenital heart disease and the following outcomes
Source: Mol Cytogenet. 2024 May 13;17:12. doi: 10.1186/s13039-024-00681-5 (PMC11089693; doi:10.1186/s13039-024-00681-5)
Supplement: Supplementary file 3 — Additional file 3. [file 13039_2024_681_MOESM3_ESM.docx]

| Table S3 The pregnancy outcome of all cases | | | | |
| --- | --- | --- | --- | --- |
| Chromosome | TOP | Delivery | χ^2^ | *P* |
| AUP or P/LP CNV | 32 | 2 | 49.031 | 0.000 |
| No or B/LB/Vous CNV | 35 | 98 |  |  |
