# Supplementary material for: Efficiency of copy number variation sequencing combined with karyotyping in fetuses with congenital heart disease and the following outcomes
Source: Mol Cytogenet. 2024 May 13;17:12. doi: 10.1186/s13039-024-00681-5 (PMC11089693; doi:10.1186/s13039-024-00681-5)
Supplement: Supplementary file 4 — Additional file 4. [file 13039_2024_681_MOESM4_ESM.docx]

| Table S4 The prognosis classification and pregnancy outcome of 133 cases | | | |
| --- | --- | --- | --- |
| Prognosis classification | n | TOP | Delivery |
| I | 100 | 7 | 93 |
| II | 8 | 3 | 5 |
| III | 14 | 14 | 0 |
| IV | 11 | 11 | 0 |
