# Supplementary material for: Efficiency of copy number variation sequencing combined with karyotyping in fetuses with congenital heart disease and the following outcomes
Source: Mol Cytogenet. 2024 May 13;17:12. doi: 10.1186/s13039-024-00681-5 (PMC11089693; doi:10.1186/s13039-024-00681-5)
Supplement: Supplementary file 5 — Additional file 5. [file 13039_2024_681_MOESM5_ESM.docx]

| Table S5 Criteria for determining copy number of detected chromosome fragments | |
| --- | --- |
| ***SCN** | **Criteria** |
| <0.84 | <1 copy |
| 0.84≤SCN≤1.16 | 1 copy |
| 1.16＜SCN＜1.78 | 1 copy and 2 copy chimerism |
| 1.78≤SCN≤2.24 | 2 copy |
| 2.24＜SCN＜2.72 | 2 and 3 copy chimerism |
| 2.72≤SCN≤3.28 | 3 copy |
| SCN＞3.28 | >3 copy |
| *SCN: The sequencing copy number of detected chromosome fragments | |
